# Challenges and constraints to the sustainability of poultry farming in Thailand

**DOI:** 10.5713/ab.24.0685

**Published:** 2025-02-25

**Authors:** Sawitree Wongtangtintharn, Sudpradthana Chakkhambang, Padsakorn Pootthachaya, Anusorn Cherdthong, Metha Wanapat

**Affiliations:** 1 Department of Animal Science, Faculty of Agriculture, Khon Kaen University, Khon Kaen, Thailand; 2 LinQ Technology Corporation, Chachoengsao, Thailand; 3 Tropical Feed Resources Research and Development Center (TROFREC), Department of Animal Science, Faculty of Agriculture, Khon Kaen University, Khon Kaen, Thailand

**Keywords:** Policy, Poultry, Production, Sustainability, Transformation

## Abstract

The poultry farming industry in Thailand plays a crucial role in the nation’s economy and food security. However, its long-term sustainability is challenged by disease outbreaks, environmental concerns, rising feed costs, market fluctuations, and shifting consumer preferences. This review examines the current state of poultry farming in Thailand, focusing on broilers, layers, and ducks. Disease control requires strict biosecurity measures and government interventions. Additionally, the industry must address environmental concerns by reducing greenhouse gas emissions and optimizing resource efficiency to align with Thailand’s net-zero targets. The rising demand for organic and ethically produced poultry products presents both market opportunities and production challenges, necessitating significant adjustments in farming practices. Regulatory compliance, particularly in export markets, adds another layer of complexity, requiring Thai poultry producers to meet increasingly stringent international standards. The adoption of new technologies, such as precision farming and artificial intelligence-driven systems, presents both opportunities and challenges, especially for smaller producers who may struggle to keep pace with advancements. Rising feed costs, driven by global supply chain disruptions and dependence on imported raw materials, pose a significant economic burden, emphasizing the need for alternative protein sources such as insect-based feed and agro-industrial by-products. Furthermore, regulatory compliance with international standards and consumer-driven trends toward antibiotic-free and organic poultry products further shape the industry’s transformation. To ensure sustainable development, Thailand’s poultry sector must integrate innovative technologies, adopt environmentally friendly practices, and strengthen collaboration between the government, industry stakeholders, and researchers. The industry can maintain its global competitiveness by addressing these challenges while contributing to sustainable food production and environmental conservation.

## INTRODUCTION

The world is currently facing challenges that threaten food security. Various factors, including economic recession and environmental change, have been identified as reasons for the decline in global food production capacity [1]. Projections suggest that the global population will reach 10 billion by 2050, leading to an increase in the demand for food [2,3]. The growing demand emphasizes the need to urgently address the issue of global hunger, which has become increasingly important in recent years. This situation presents a significant challenge to the achievement of the Sustainable Development Goals (SDGs), specifically aimed at eliminating hunger by 2030 [4,5].

Thailand’s role in the global poultry market is noteworthy, ranking as the seventh-largest poultry meat producer and the fourth-largest exporter [6,7]. Over the past ten years, the nation’s poultry production has seen a consistent annual growth of 3.59%, with consumption rising at a rate of 6.94% [8]. Concurrently, there has been a notable increase in poultry meat exports. Thailand’s poultry industry, a vital component of its agricultural sector, faces a complex interplay of challenges and opportunities as it strives for sustainability. Thailand’s poultry farming industry, encompassing broiler, layer, and duck production, is a vital sector that significantly contributes to the nation’s economy and food security. While the industry has demonstrated resilience and adaptability, addressing issues such as disease outbreaks, environmental concerns, and market fluctuations are crucial for its long-term viability [9].

Disease outbreaks, particularly avian influenza, have posed significant threats to the industry, requiring robust biosecurity measures and government interventions. Environmental sustainability is another pressing concern, with the industry needing to balance economic growth with minimizing its ecological footprint [10,11]. Rising feed costs and raw material dependencies present additional challenges, affecting profitability and competitiveness. Similarly, feed costs (e.g., soybean meal, maize) remain a significant burden, as the industry imports much of its raw material, making it vulnerable to global supply chain disruptions.

Moreover, the industry must navigate evolving consumer preferences and market dynamics, including growing demands for organic, high-protein, and environmentally friendly products. Adapting to these changing trends while maintaining profitability requires innovative approaches and continuous improvement [12,13]. To address these challenges and ensure the sustainability of the poultry industry, Thailand has implemented various strategies. Government initiatives focus on strengthening biosecurity measures, promoting sustainable farming practices, and supporting research and development. The private sector is also actively involved, investing in innovative technologies and adopting sustainable production methods.

Despite these efforts, the poultry industry still faces significant obstacles. Disease outbreaks remain a constant threat, and the industry must continue to invest in prevention and control measures [3]. Environmental sustainability requires significant changes in production practices, including reducing greenhouse gas (GHG) emissions, optimizing resource use, and adopting sustainable feed formulations. The environmental impact of large-scale poultry farming is another pressing concern. With Thailand committed to reducing its GHG emissions and achieving net-zero targets by 2065, the poultry sector is under pressure to adopt more sustainable practices. These include reducing CO_2_ emissions, improving resource efficiency, and minimizing waste [13]. Moreover, consumer demands for organic and environmentally friendly products add another layer of complexity, requiring the industry to innovate while balancing cost and production efficiency [12,14]. Furthermore, the industry must navigate complex regulatory frameworks, both domestically and internationally, to ensure compliance and maintain market access. Balancing these various challenges while maintaining profitability and competitiveness requires a multifaceted approach that involves collaboration between government, industry, and academia.

By addressing these challenges and embracing sustainable practices, Thailand’s poultry industry can position itself for long-term success. This will not only contribute to the country’s economic growth and food security but also demonstrate its commitment to sustainable agriculture and environmental stewardship. Balancing economic viability with environmental responsibility is key to ensuring the long-term success of Thailand’s poultry industry, particularly as it navigates both domestic demands and international market pressures.

## CURRENT STATUS OF THE POULTRY PRODUCTION INDUSTRY IN THAILAND

The poultry industry in Thailand, which encompasses the production of broiler chickens, laying hens, and ducks, is a critical sector that plays a significant role in driving the economy and ensuring food security domestically and internationally. While the industry has demonstrated resilience and adaptability, it still faces challenges and issues that must be addressed to achieve sustainability. These include disease outbreaks, environmental concerns, fluctuations in raw material prices and quantities, and market volatility, which are crucial for long-term sustainability [15]. Additionally, the poultry industry must adapt to changing consumer preferences, with health-conscious consumers increasingly opting for organic, chemical-free products and environmentally friendly options [11,12]. To keep up with these evolving trends, Thailand’s poultry industry must continuously innovate and improve animal production methods to meet challenges and ensure the industry’s sustainability. Besides private sector investments in new technologies and sustainable production methods, the government has policies to strengthen biosecurity measures, promote sustainable farming practices among farmers, and support research and development for relevant agencies.

Thailand has three types of poultry farms: large private companies, contract farming, and small-scale farmers. These farms operate and produce according to the guidelines of international standards such as good hygiene practices (GHPs) and hazard analysis critical control point (HACCP). The production process is fully integrated, covering farms, feed mills, slaughterhouses, and food product processing that adhere to these standards. Additionally, they follow other standards such as the International Organization for Standardization (ISO) and the Feed Additive and Pre-mixture Quality System. These measures ensure that many countries have confidence in the poultry products that come from Thailand.

### Broiler production industry in Thailand

**Figure 1:**
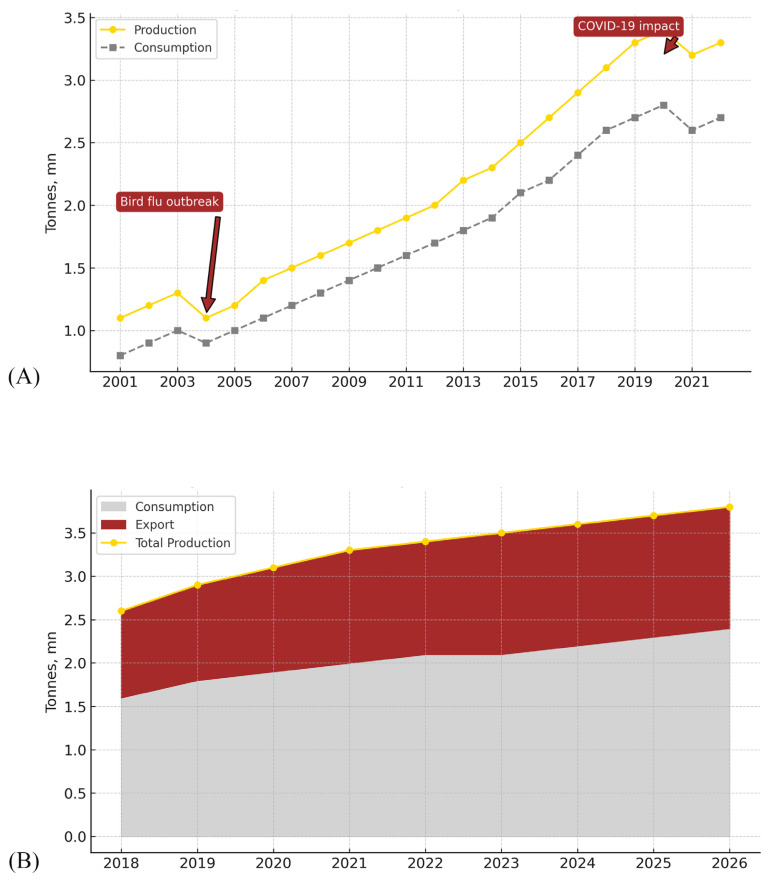
Thai chicken production, (A) consumption volume, and (B) export volume. mn, million. Data from Kornboontritos [14].

The broiler production industry in Thailand plays a crucial role in the country’s agricultural sector and economy. It significantly impacts Thailand’s gross domestic product, provides employment, ensures food security, and generates export revenues. Following the 2004 avian influenza outbreak, broiler farms in Thailand transitioned to closed systems and adopted compartmentalization methods. This adaptation significantly reduced disease outbreaks, and Thailand has not experienced any outbreaks since 2007 [16]. This change allowed Thailand to continuously increase its broiler meat exports, making it the world’s sixth-largest exporter of frozen chicken in 2021. During the COVID-19 pandemic, poultry production and consumption increased due to reduced consumer purchasing power and the cost-effectiveness of chicken. As a result, Thailand’s export volume grew by 4.4% in 2023 (Figures 1A, 1B).

**Figure 2:**
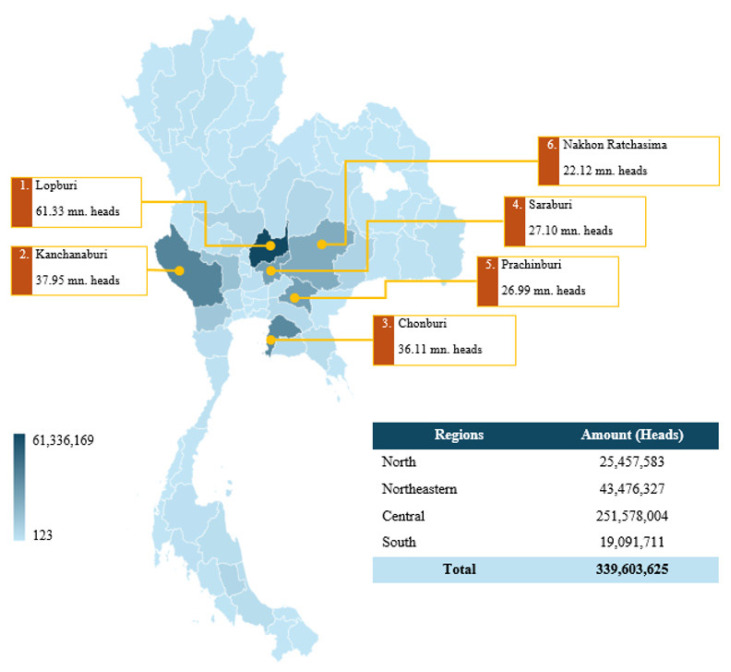
Map showing the density of broiler chicken farming in Thailand, 2024. mn, million. Data from Department of Livestock Development [19].

Over the past year, there have been 31,117 broiler producers in Thailand [17]. A comprehensive tally of 12,567 commercial broiler farms has obtained accreditation in accordance with good agricultural practice standards. The annual production is approximately 1,927 million birds. Most farmers nationwide have small-scale broiler farms, with a maximum capacity of 20,000 broilers per farm. On the contrary, large farms have the capacity to simultaneously rear a range of 100,000 to 1,000,000 broilers [17,18]. According to the most recent data from the Department of Livestock Development (September 2024), the majority of broiler-raising sources are located in the central region, which accounts for 74.1% of all chicken produced in Thailand, followed by the northeastern region (12.8%), the northern region (7.5%), and the southern region (5.6%). By individual province, Lopburi is the leading source of broiler production, contributing 18.1% of the national chicken output, followed by Kanchanaburi (11.2%), Chonburi (10.6%), Saraburi (8.0%), and Prachinburi (7.9%) (Figure 2) [19].

**Figure 3:**
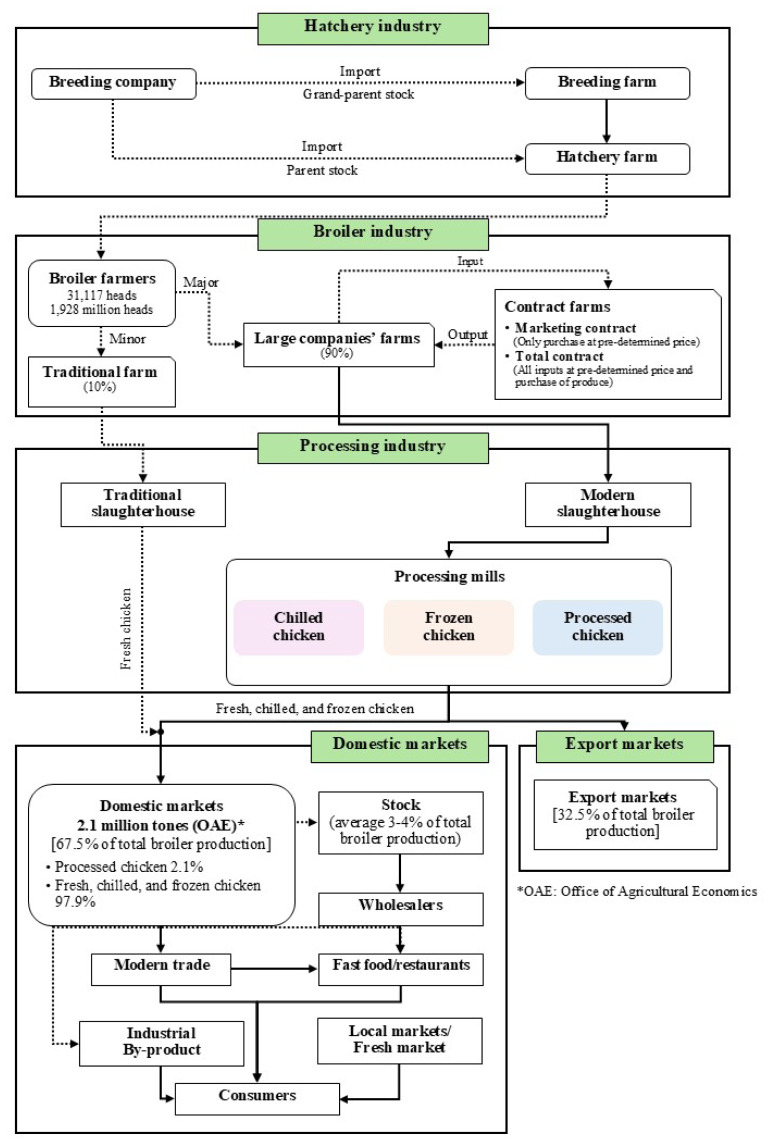
The supply chain of chicken industry in Thailand, 2022. Data from Kornboontritos [14].

In Thailand, broiler farms primarily operate as integrated businesses, which include parent stock farms, hatcheries, broiler farms, feed mills, slaughterhouses, and processing plants that meet safety standards. The farms are categorized into large companies (50%), contract farms (40%), and traditional farms (10%). Traditional poultry farms primarily supply chickens to small-scale slaughterhouses for domestic consumption. In 2022, broiler production reached approximately 1.93 billion birds [14]. The majority of production (90%) is controlled by large poultry companies, which manage integrated operations across the supply chain, including animal feed production, broiler farming (through both company-owned farms and contract farming systems), slaughterhouses, and food processing facilities that adhere to recognized industry standards. This vertically integrated structure provides large corporations with cost efficiency and economies of scale, allowing them to maintain competitive pricing and operational stability. In contrast, small-scale poultry farms account for only 10% of total production and rely almost exclusively on the domestic market (Figure 3).

**Figure 4:**
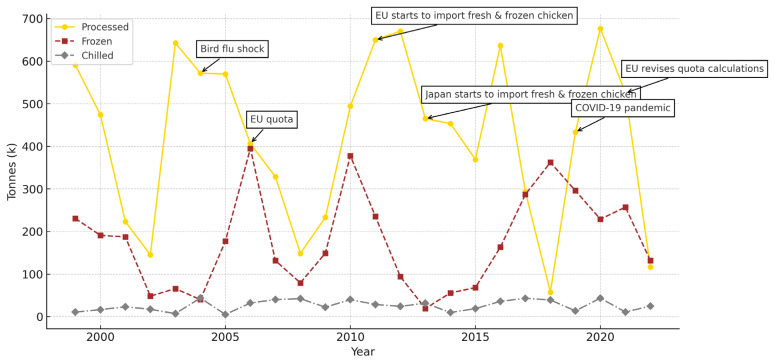
Quantity of chicken exported from Thailand, 1999–2022. Data from Kornboontritos [14].

The Bank of Ayudhya Public Company Limited looked at the trends of broiler products from 2024 to 2026 and found that 60% to 70% of the meat produced is used in India and the other 30% is sent abroad. Domestic products mainly include chilled chicken, frozen chicken, and processed or cooked chicken, while export products primarily consist of processed chicken—accounting for 64.5% of all chicken product exports from Thailand (Figure 4). The main markets for processed chicken are Japan (47.7% of all processed chicken exports), the United Kingdom (26.5%), the Netherlands (8.6%), and South Korea (4.8%). Frozen chicken constitutes 35.0% of Thailand’s chicken exports, with key destinations being Japan (38.4% of all frozen chicken exports), China (24.1%), Malaysia (20.4%), and South Korea (3.4%). Chilled chicken accounts for only 0.5% of Thai chicken exports, with primary markets located in neighboring or nearby countries. Myanmar is the largest importer, taking 61.0% of these exports, followed by Hong Kong (21.5%), Cambodia (7.1%), and Singapore (6.9%) [14].

The economists forecast that from 2024 to 2026, broiler meat production in Thailand will increase by an average of 1.5% to 2.5% per year, with domestic and international sales continuing to rise. However, the industry faces challenges, including high production costs compared to competitor countries, geopolitical conflicts, and the need to expand trade markets. The avian influenza outbreak in Brazil presents an opportunity for Thai exports, but the industry must navigate complex global market dynamics to maintain its competitive edge.

### Layer hen production industry in Thailand

**Figure 5:**
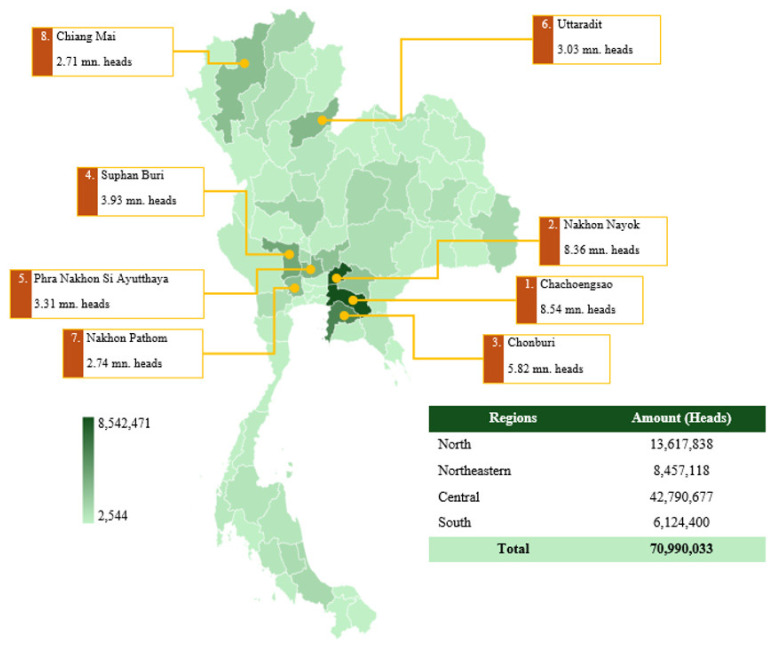
Map showing the density of laying chicken farming in Thailand, 2024. mn, million. Data from Department of Livestock Development [19].

The number of laying chickens in the country as of September 2024 is 70.99 million heads, with production density varying by region (Figure 5). The central region remains the primary hub for broiler production, accounting for 42.79 million heads (60.3%) of the national total. This is followed by the northern region with 13.62 million heads (19.2%), the northeastern region with 8.46 million heads (11.9%), and the southern region with 6.12 million heads (8.6%). The highest-producing provinces include Chachoengsao (8.54 million heads), Nakhon Nayok (8.36 million heads), and Chonburi (5.82 million heads), highlighting the dominance of the central and eastern parts of Thailand in commercial broiler farming. The density of broiler farms varies significantly across the country, with provinces such as Suphan Buri (3.93 million heads), Phra Nakhon Si Ayutthaya (3.31 million heads), and Uttaradit (3.03 million heads) also playing a critical role in poultry production. The northern provinces, including Chiang Mai (2.71 million heads) and Nakhon Pathom (2.74 million heads), contribute considerably to the overall supply [19].

**Figure 6:**
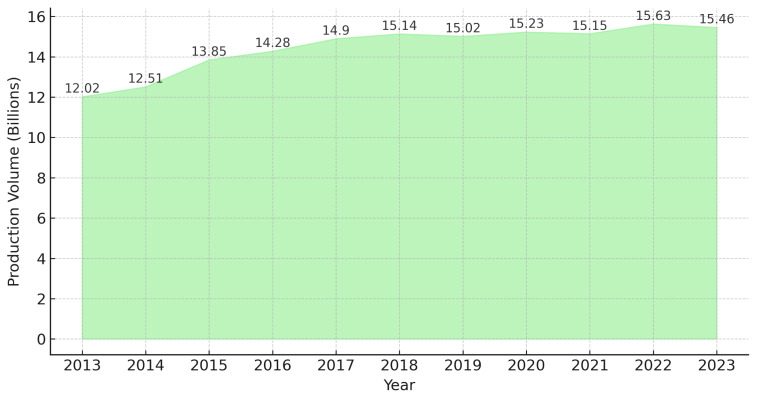
Production volume of hen eggs in Thailand from 2013 to 2023. Data from Statista [23].

The egg production industry of Thailand consists of three main farm types: traditional farms, contract farms, and large-scale commercial farms [20]. Contract and large-scale farms typically use closed systems with caged housing [21]. Currently, Thailand’s layer production industry has demonstrated resilience and adaptability between 2022 and 2025. In 2022, the country exported eggs valued at $35.4 million, ranking as the 28th largest egg exporter worldwide. The primary export destinations included Singapore, Hong Kong, and Cambodia. Conversely, imports were minimal, totaling $1.6 million, with Malaysia and Italy as the main suppliers [22]. This trade balance highlights Thailand’s self-sufficiency in egg production, ensuring stable domestic supply while maintaining a presence in international markets. Meanwhile, in 2023, the country produced approximately 15.46 billion chicken eggs, reflecting a slight decrease from previous years (Figure 6). This decline has been attributed to environmental challenges, which negatively affected poultry health and egg production rates. Despite these challenges, Thailand remains a strong player in the global egg market.

Thailand’s egg production primarily supports domestic consumption, with 95% of output meeting local demand, while 5% is exported to Singapore, Hong Kong, Taiwan, and Malaysia. In recent years, production has remained stable due to government regulations on breeder layer imports, managed by the Egg Board. This strategic regulation helps control production levels and stabilize domestic egg prices. However, in 2023, egg prices exhibited significant volatility due to increased exports driven by overseas avian influenza outbreaks, which led to domestic shortages and price fluctuations. Analysts predict that Thailand’s egg export capacity will increase in 2024, further reinforcing its position in the global egg market.

Looking ahead, the market outlook remains optimistic. Projections indicate that Thailand’s egg market will generate approximately $960 million in revenue by 2025, with an anticipated annual growth rate of 4.91% from 2025 to 2030 [23]. This growth is expected to be driven by rising consumer health awareness and increasing demand for organic and free-range eggs. Additionally, the food service sector is shifting toward cage-free eggs and sustainable farming practices to meet evolving consumer preferences, reinforcing a broader commitment to ethical sourcing and animal welfare. Meanwhile, Thailand’s layer production industry has faced environmental challenges leading to a slight production decline in 2023; it continues to play a crucial role in the global egg market. The country’s self-sufficiency, strategic export relationships, and positive market outlook, supported by consumer-driven demand for high-quality and ethically sourced eggs, position it for continued growth and sustainability in the coming years.

### Duck production industry in Thailand

The duck production and distribution industry in Thailand operates as a midstream to downstream agribusiness sector within the integrated duck farming industry. Most businesses procure ducks from contract farmers (contract farming), while some also invest in closed-system duck farms to ensure a stable supply. Ducks in Thailand are classified into two primary categories, including meat-type ducks and egg-type ducks, each serving distinct commercial purposes. Meat-type ducks serve as the primary raw material for duck meat production. The most widely farmed breed is the Pekin duck, known for its high meat yield and body weight. Another notable breed is the Muscovy duck, which grows larger but at a slower rate than the Pekin duck; it is often crossbred with other duck breeds to accelerate growth. Additionally, free-range meat ducks are raised in rice fields by small-scale farmers to control pests. However, this method accounts for a smaller proportion of total production compared to commercial closed-system farming, which is primarily used for large-scale trade. Egg-type ducks are bred specifically for egg production. Once they reach approximately two years of age, they are culled and sold for meat. Similar to meat ducks, egg ducks are raised using two distinct farming methods: industrial closed-system farming for large-scale production and free-range farming, where ducks are allowed to forage in open fields, particularly in rice paddies.

**Figure 7:**
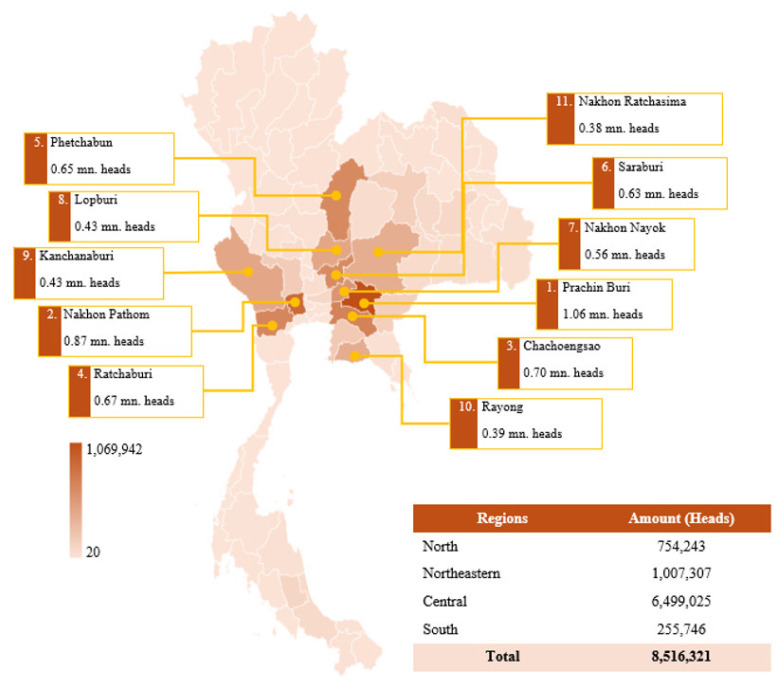
Map showing the density of meat-type duck farming in Thailand, 2024. mn, million. Data from Department of Livestock Development [19].

The amount of meat-type duck population in the country, as of September 2024, is approximately 8.52 million heads, with the central region serving as the primary hub for production. The central region accounts for 6.50 million heads (76.3%), followed by the northeastern region (11.8%), the northern region (8.9%), and the southern region (3.0%). Among individual provinces, Prachin Buri (1.06 million heads), Nakhon Pathom (0.87 million heads), and Chachoengsao (0.70 million heads) represent the largest duck-producing provinces, highlighting the dominance of the central region in duck farming. Conversely, the southern region has the lowest duck population (Figure 7) [19].

**Figure 8:**
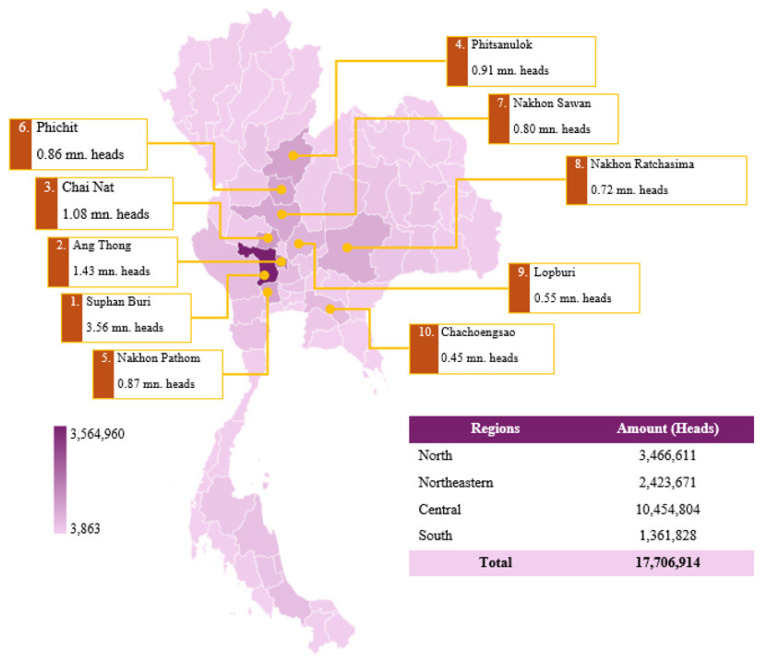
Map showing the density of egg-type duck farming in Thailand, 2024. mn, million. Data from Department of Livestock Development [19].

Egg-type duck farming in Thailand is characterized by a combination of traditional smallholder farms and commercial-scale production systems. Traditional farms often utilize open-range systems, where ducks are allowed to forage in rice fields, while commercial farms employ intensive rearing systems with controlled environments and modern feeding practices to enhance egg productivity and quality. As of September 2024, the total duck population raised for egg production is 17.71 million heads, with the central region serving as the primary hub for production. The central region accounts for 10.45 million heads (59.1%), followed by the northern region (19.6%), the northeastern region (13.7%), and the southern region (7.7%) (Figure 8). Among individual provinces, Suphan Buri (3.56 million heads), Ang Thong (1.43 million heads), and Chai Nat (1.08 million heads) are the leading duck egg-producing provinces, highlighting the central region’s dominance in the industry. Other key production areas include Phitsanulok (0.91 million heads) and Nakhon Sawan (0.80 million heads) in the northern region, as well as Nakhon Pathom (0.87 million heads) in the central region [19]. The northeastern and southern regions contribute lower production volumes, reflecting differences in regional farming practices, climate conditions, and market demand.

The duck farming situation in 2024 recorded a total of 26.2 million ducks, reflecting an 18% decrease from 2022. This decline contrasts with the average annual growth rate of 3.3% observed between 2018 and 2022. In particular, the number of meat-type ducks in 2024 stood at 8.5 million, marking a sharp decline of 42% compared to the previous year [24]. This drop was primarily due to farm closures, with the majority being small-scale duck meat farmers. These farmers were significantly affected by inflation, which resulted in their inability to bear the increased costs of animal feed and energy, making it difficult for them to sustain operations.

**Figure 9:**
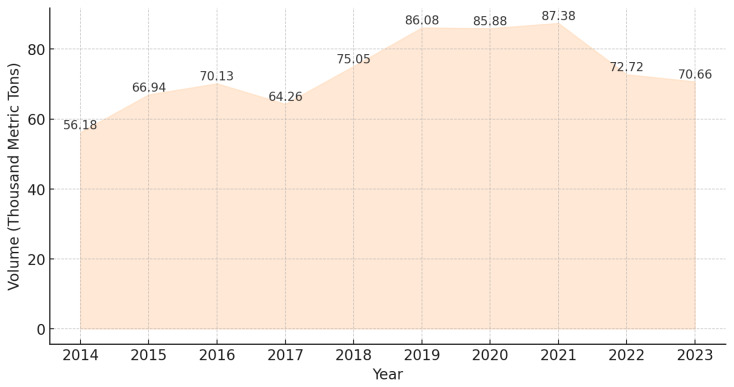
Production volume of duck meat in Thailand from 2014 to 2023. Data from Statista [25].

Between 2014 to 2016, Thailand’s duck meat production experienced steady growth, increasing from 56.18 to 70.13 thousand metric tons. This growth was driven by improvements in breeding techniques, farm expansion, and rising demand. However, in 2017, production declined to 64.26 thousand metric tons, likely due to rising feed costs and disease-related challenges. A strong recovery followed in 2018, with production reaching 75.05 thousand metric tons, continuing into 2019, when it peaked at 86.08 thousand metric tons, supported by higher export demand and increased investment in commercial duck farming. Between 2019 and 2021, production remained relatively stable, reaching its highest point at 87.38 thousand metric tons in 2021, despite economic challenges caused by the COVID-19 pandemic. However, in 2022, production declined to 72.72 thousand metric tons (Figure 9) [25], likely due to supply chain disruptions and rising production costs.

Thailand’s duck production industry continues to face challenges and shifts in 2023 to 2024. Recent data indicates a more than 5% decrease in overall duck production, with duck meat output dropping by 2.8% to 70,660 tons in 2023 (Figure 9) [24]. Rising feed costs, surpassing the selling price of duck products, are primarily responsible for this decline, thereby reducing farmers’ profitability. Additionally, with higher feed and energy costs, coupled with an increase in chicken egg prices, consumers have shifted toward consuming duck eggs instead. As a result, some farmers have switched to raising free-range egg ducks, which have lower production costs compared to other duck farming systems.

Meanwhile, domestic consumption of duck meat has declined, primarily due to a 10% to 15% increase in fresh duck wholesale prices, driven by higher feed costs, while chicken meat prices have decreased, making it a more affordable alternative. Due to these factors, analysts predict that domestic duck meat consumption will stabilize in 2024. On the export front, a slight expansion is expected, particularly in cooked duck meat exports to Australia and Japan. This trend suggests that while the domestic market faces pricing and cost challenges, the international market offers potential opportunities for growth in Thailand’s duck meat industry.

## THE CHALLENGES OF DEVELOPING THAILAND’S POULTRY INDUSTRY TOWARD SUSTAINABILITY

### Disease outbreaks

Disease outbreaks, particularly highly pathogenic avian influenza, pose significant challenges to the poultry industry. In 2004, Thailand faced an avian influenza outbreak, leading to the suspension of broiler meat exports. However, the public and private sectors swiftly resolved the issue by revamping poultry farming systems and implementing biosecurity measures. Producers in the food production chain were required to follow food safety standards such as GHPs and HACCP. Additionally, the government enforced strict border control measures, including the establishment of quarantine units and patrols by border police to prevent the entry of diseases. As a result of these efforts, Thailand has remained free of avian influenza since 2007, leading to a 26% increase in broiler meat exports as of 2021 [14].

In addition to surveillance of disease outbreaks, the livestock production industry must closely monitor pathogenic microorganisms such as *Salmonella*, *Coccidiosis*, *Clostridium*, and *E. coli*. These pathogens can negatively impact animal growth, health, and the quality of food products. Strict control and prevention measures must be applied at every stage, from animal farms and feed mills to food processing plants. While these pathogens can be eliminated, collaboration among all stakeholders in the production chain, from upstream to downstream, is required. The rigorous implementation of biosecurity standards is essential to ensure animal health and maintain product quality, preventing any threats from these microorganisms [26].

Furthermore, the widespread use of antibiotics in food-producing animals has raised concerns as it contributes to the development of antibiotic-resistant bacteria, which can potentially be transmitted to humans [27]. In recent years, the global rise of antibiotic resistance has intensified scrutiny over the administration of antibiotics in livestock production [28,29]. The risks associated with antibiotic use extend beyond the emergence of resistant zoonotic bacteria and veterinary pathogens, as there is also the possibility of antibiotic residues remaining in animal-derived food products [27]. In response to these challenges, the World Organization for the Humane Treatment of Animals has initiated strategies aimed at minimizing the emergence of drug resistance by exploring innovative approaches to animal welfare management. As a result, poultry producers across various countries have begun integrating these principles into their production systems to promote responsible antibiotic use and sustainable farming practices [30].

As the fourth largest broiler exporter globally [31], Thailand’s poultry producers must adhere to strict regulations and standards set by importing countries. Compliance with animal welfare practices and the elimination of antibiotic use in broiler production is particularly crucial for exports to Europe [32]. To meet these requirements, Thai broiler producers aiming to access European markets must operate under the Raised Without Antibiotics (RWA) program, which prohibits the use of antibiotics in feed, water, or injections. However, to manage coccidiosis, producers are permitted to use chemical coccidiostats, while alternative solutions such as essential oils and probiotics can serve as substitutes for antibiotics in maintaining poultry health [33]. If broilers require antibiotic treatment due to illness, they are removed from the RWA program and must be directed to alternative distribution channels. Furthermore, Thai broiler producers committed to antibiotic-free and animal welfare-compliant farming must carefully manage risk factors throughout rearing, pre-processing, transportation, and slaughter, as these stages significantly impact the economic viability of broiler production.

### Regulatory standards and policies, climate change, and environmental

Climate change is inevitable and is an issue of global concern. The impacts of climate change are happening, and they extend well beyond an increase in temperature, affecting ecosystems, human health, economics, and social systems in all regions around the world, as well as the productivity of livestock species. While the poultry industry is affected by climate change, it also contributes to GHG emissions, necessitating mitigation strategies. This is further exacerbating environmental pressures and necessitating urgent mitigation strategies.

The United Nations has called for global cooperation from businesses to join agreements to reduce GHG emissions. Mitigating GHG emissions has become a critical environmental priority for developing nations. In response to climate change challenges, Thailand has implemented several strategic measures to minimize its environmental impact. These include commitments under the Nationally Appropriate Mitigation Action framework, the development of a Nationally Determined Contribution (NDC) roadmap, and initiatives to enhance resilience across all sectors. As part of its global climate commitments, Thailand submitted its Long-Term Low Greenhouse Gas Emissions Development Strategy to the United Nations Framework Convention on Climate Change (UNFCCC) in 2021 [34], followed by a revised version in November 2022 [35]. This strategic framework outlines key mitigation policies and enhanced reduction efforts, with the goal of achieving carbon neutrality by 2050 and net-zero GHG emissions by 2065. To reach its goals of net-zero emissions and carbon neutrality by 2050, Thailand updated its NDC by raising the target for reducing GHG emissions from 25% to 40% by 2030 [36]. It may need to speed up this process to meet the rules of the European Union (EU), which is still an important market for food exports. To maintain Thailand’s competitive edge and export opportunities to Europe, businesses must urgently implement GHG reduction measures in line with EU requirements. This is crucial to avoid future impacts on Thailand’s export sector.

The livestock sector was initially perceived as a passive contributor to climate change, but it is now recognized as a major source of GHG emissions, including carbon dioxide (CO_2_), methane (CH_4_), and nitrous oxide (N_2_O). The carbon footprint of an industry, enterprise, or activity is determined by measuring its GHG emissions. Research analyzing the environmental impact of various food products found that broiler production generates 9.87 kg of CO_2_-equivalent emissions per kilogram of meat, which is lower than emissions from cattle (both beef and dairy herds), lamb, mutton, and pork production [37]. Additionally, poultry farming contributes approximately 8% of total emissions from the livestock sector, equating to 0.6 gigatons of CO_2_-equivalent emissions [38]. These findings highlight the potential of choosing lower-emission protein sources, such as broilers or layers, to reduce the overall carbon footprint of food production, thereby supporting climate change mitigation efforts.

### Consumer and market demand

Developing Thailand’s poultry industry toward sustainability requires addressing evolving consumer preferences and market demands, which present both challenges and opportunities for producers. One of the key drivers of change is increasing consumer awareness regarding food safety, animal welfare, and environmental impact. Modern consumers, particularly in export markets such as the EU, Japan, and Australia, demand poultry products that meet higher standards for antibiotic-free, organic, and sustainably produced meat and eggs.

Another challenge lies in the market shift towards protein diversification, where plant-based and cultured meat alternatives are gaining popularity. While poultry remains one of the most consumed proteins globally, the rise of lab-grown and plant-based proteins is influencing consumer behavior. To stay competitive, Thailand’s poultry industry must innovate, focusing on high-quality, traceable, and value-added poultry products, such as fortified eggs, functional poultry meat, and sustainable protein solutions. For Thailand’s poultry industry, this presents both opportunities and challenges. While there is potential to access new market segments, significant adaptation in production methods, marketing strategies, and product development is required to meet these sophisticated consumer expectations [12,26].

Moreover, export regulations and trade barriers continue to shape Thailand’s poultry industry. As sustainability becomes a key requirement for market access, importers in the EU and other developed countries are implementing stricter carbon footprint regulations, requiring Thai poultry producers to reduce GHG emissions and adopt environmentally friendly practices. The challenge lies in balancing sustainability goals while maintaining cost-efficiency and price competitiveness in both domestic and international markets.

### New technology and innovation

being adopted to enhance production efficiency through collaboration among government agencies, universities, and the private sector. For example, clustered regularly interspaced short palindromic repeats technology is a genome-editing tool that enables precise modifications to DNA, such as adding, removing, or replacing sequences. This technology can enhance traits in poultry, including disease resistance and growth efficiency [39]. Advanced precision farming systems, machine learning for weight forecasting, generative artificial intelligence, and the internet of things are pivotal in enhancing production efficiency. They enable real-time monitoring and recording of critical factors such as chicken weight, environmental conditions (temperature and humidity), and feed and water consumption. This real-time data allows managers to make more informed decisions, optimize conditions, and improve overall management practices, leading to increased efficiency and productivity in poultry farming.

However, limited access to training and technical knowledge about modern poultry farming techniques and their adoption has continued to be a challenge for small- and medium-scale farmers. The transition towards sustainable poultry production in Thailand is highly dependent on new technology and innovation, yet adopting these advancements presents several challenges. One of the most pressing issues is the high cost of investment in modern technologies, potentially leading to a widening gap between large commercial farms and smaller producers.

Furthermore, the push for sustainable innovations in feed production also presents both opportunities and obstacles. The use of alternative protein sources such as insect-based feed, algae, and fermented proteins can reduce reliance on soybean and fishmeal [40,41], which have high environmental footprints. However, regulatory approval, consumer acceptance, and cost-efficiency remain challenges in scaling up these innovations. The information presented highlights that the lack of cost of investment, technical knowledge, and skills has hampered productivity and limited the ability to adopt improved farming techniques, hence decreasing productivity and efficiency.

### Feed and raw material costs

Feed cost remains a major challenge for Thailand’s poultry industry, primarily due to its heavy reliance on imported feed ingredients such as corn, soybean meal, and fish meal. Domestic production is insufficient to meet demand, necessitating large-scale imports. The annual demand for feed corn is approximately 8 to 8.5 million tons, while domestic production only reaches 4.9 million tons, leading to imports of around 3 million tons [42]. Similarly, soybean meal demand stands at 4 to 4.2 million tons per year, but local production supplies only 2 million tons, requiring imports of about 2.2 million tons [43]. Additionally, fish meal consumption is estimated at 600,000 tons annually, yet domestic production meets only 300,000 tons, creating a continued need for imports [42]. These imported raw materials are crucial for maintaining the stability and sustainability of Thailand’s livestock and animal feed industry. Furthermore, feed ingredient prices fluctuate based on production volume, fuel costs, and transportation expenses, making feed costs in Thailand significantly higher than in feed-producing countries like Brazil and the USA.

In recent years, the Ukraine-Russia crisis has disrupted global grain markets, impacting the availability and affordability of imported feed ingredients [44]. This has contributed to feed shortages, increased feed costs, and reduced agricultural productivity. These crises underscore the interconnectedness of global and regional factors that shape the challenges faced by Thailand’s poultry farmers in securing an adequate and cost-effective supply of feed ingredients. As a response, there is growing potential to expand the use of agro-industrial by-products as alternative feed sources to alleviate feed scarcity issues. Addressing this challenge requires the development and exploration of alternative feed ingredients that can replace or supplement primary feed sources. Creating low-cost, high-nutritional-value alternatives will be crucial in reducing feed costs and enhancing the competitiveness of Thailand’s poultry industry.

**Figure 10:**
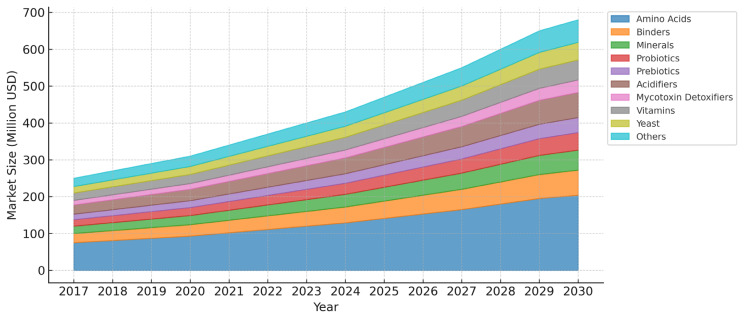
Value of feed additive by categories (USD), Thailand, 2017–2030. Data from Mordor Intelligence [45].

The current situation of the feed additives market in Thailand witnessed significant growth in 2022, with amino acids, binders, minerals, and probiotics playing a crucial role in compound feed production, accounting for 55.8% of the total market share. Among these, methionine and lysine were the most widely used amino acids, contributing 30.8% and 21.8% of the market value, respectively (Figure 10) [45]. These additives are favored for their efficiency in improving gut health, enhancing digestion, and supporting meat production. Additionally, synthetic feed binders were widely used to improve digestion and nutrient absorption, reducing disease risks. These binders dominated the feed binder market, accounting for 63.8% of the total market share in 2022.

In terms of livestock feed, swine production accounted for the largest share of the feed additives market at 57.2% by value, followed by poultry, which had a higher feed intake, resulting in an estimated 12.2 million metric tons of production in 2022. The rising demand for livestock production in Thailand has driven the expansion of the feed additives market, with amino acids, prebiotics, and probiotics expected to be the fastest-growing segments. Amino acids are projected to grow at a compound annual growth rate of 6.1%, while prebiotics and probiotics are expected to grow at 6% each during the forecast period [45]. Amino acids are crucial for protein synthesis, meat production, and milk production, contributing to their high growth rate and increasing adoption in Thailand’s feed industry. By integrating feed additives and alternative feed sources, Thailand’s poultry sector can improve feed efficiency, reduce reliance on costly imports, and enhance overall sustainability. This will be essential in ensuring the long-term sustainability and competitiveness of Thailand’s poultry industry.

## THE GROWING AWARENESS OF SUSTAINABILITY IN THE POULTRY PRODUCTION INDUSTRY IN THAILAND

The sustainability of poultry farming in Thailand faces several challenges, the majority related to disease outbreaks and biosecurity risks, environmental impact and climate change, market and consumer demand, technology and innovation barriers, and feed and raw material costs. To successfully transition toward sustainable poultry production, Thailand must focus on enhancing supply chain transparency, improving sustainability certifications, and adopting precision farming techniques to reduce environmental impact and meet consumer demands. Collaboration between government agencies, industry stakeholders, and research institutions will be crucial in navigating these challenges and ensuring that Thailand remains a competitive and sustainable poultry producer on a global scale.

One of the major concerns is the carbon footprint of poultry production, particularly GHG emissions, including CO_2_, CH_4_, and N_2_O. N_2_O, with a global warming potential 265 times higher than CO_2_, poses a significant environmental threat [46]. The poultry sector must find ways to reduce emissions while maintaining production efficiency. Thailand’s poultry industry is actively working toward sustainability, with efforts from both the public and private sectors. Currently, the government is drafting the Climate Change Act and the Clean Air Act, which are scheduled to be completed by 2024. [47] These laws aim to promote the reduction of GHG emissions and improve air quality. The Department of Livestock Development is collaborating with the private sector to develop sustainable use of raw materials, which is crucial for reducing costs and environmental impact. Furthermore, the Department of Livestock Development promotes the concept of “One Health,” which emphasizes the poultry industry’s holistic approach to sustainability. This framework acknowledges the interconnection of animal, human, and environmental health, aligning with the United Nations’ SDGs. This concept focuses on precision feed formulation techniques to reduce environmental impact while maintaining productivity efficacy [10,48].

One of the key sustainability concerns is feed source and feed efficiency, as poultry farming relies heavily on imported raw materials such as corn, soybean meal, and fish meal, making it vulnerable to price volatility and supply chain disruptions. To address this, Thailand is exploring alternative protein sources, including insect-based feeds, algae, and agro-industrial by-products, to reduce reliance on traditional feed ingredients and lower the industry’s environmental footprint [40,41,49]. Furthermore, feed efficiency is another crucial issue in sustainable poultry farming. The cost and availability of dietary energy and protein sources significantly impact production costs. Least-cost feed formulation and precision nutrition strategies are essential to optimizing feed conversion ratios, digestibility, and net nutrient utilization. Additionally, gut health plays a pivotal role in maximizing feed efficiency, reducing metabolic stress, and minimizing nutrient waste [50]. Poor feed utilization results in excessive excretion of pollutants, contributing to environmental degradation.

Another key constraint is energy utilization and waste management. Poultry production involves significant energy losses due to heat increment, fecal loss (8% to 25%), and urinary loss (>40%) [51]. Strategies to improve energy efficiency include the use of emulsifiers, enhanced feed formulation, and precision feeding techniques. Additionally, reducing nitrogen excretion through improved protein retention ratios can minimize environmental pollution and promote sustainability. The above guidelines are consistent with the policy of the Thai government, which have developed the Bio-Circular-Green (BCG) economic model strategy to drive the country’s economic and social development. This model focuses on the bioeconomy, or the efficient and sustainable use of biological resources; the circular economy, which promotes the optimal use of resources and reduces waste; and the green economy, which supports development that reduces energy consumption and GHG emissions. Some small-scale farmers have adopted the BCG model in poultry farming to reduce GHG emissions and promote sustainable production. Notable examples include organic eggs, which some farmers have developed as a product popular in the health-conscious market. The Department of Livestock Development has certified them as a model farm. Charoen Pokphand Foods (CPF) company has implemented the BCG model in its poultry industry, producing carbon-free eggs under the U-Farm brand [52]. The BCG project positively impacts Thailand’s egg poultry industry by promoting economic and environmental sustainability. Enhancing product value also provides a competitive edge in the global market.

By 2050, the need for poultry products is predicted to quadruple globally, and to overcome these challenges, Thailand’s poultry industry must focus on enhancing feed efficiency, reducing emissions, improving gut health, and optimizing energy utilization. Implementing innovative feeding strategies, adopting environmentally friendly production practices, and utilizing advanced nutrition technologies are crucial steps toward ensuring the long-term sustainability of poultry farming in Thailand.

## CONCLUSION

The poultry industry in Thailand faces several challenges in achieving future sustainability goals, such as sourcing alternative raw materials to reduce dependency on imported raw materials by developing cost-effective and sustainable alternatives such as insects, algae, or by-products from food processing. Developing appropriate feed formulations that enhance growth performance, reduce antibiotic use, and minimize waste. Using additives like enzymes, organic acids, probiotics, and prebiotics to improve nutrient utilization and digestion efficiency. Investment in adopting new technologies and innovations such as smart farm systems, temperature and humidity control, and alternative energy sources. Large companies can invest, but medium and small-scale farmers face investment challenges. Environmental challenges include reducing GHG emissions and enhancing sustainability. Support is needed to encourage research and development of technologies and innovations aimed at sustainability. This includes supporting the development of new technologies that can be practically applied in industry and disseminating knowledge. Training farmers and industry operators to highlight the importance of sustainable practices and GHG reduction-building collaboration among government agencies, the private sector, and research organizations to share knowledge and resources for developing new feed formulations and technologies. Addressing these challenges will help Thailand advance its poultry industry towards sustainability and achieve net-zero goals effectively.

## References

[1] Liadze I, Macchiarelli C, Mortimer-Lee P, Sanchez Juanino P (2023). Economic costs of the Russia-Ukraine war. World Econ.

[2] Nhemachena C, Nhamo L, Matchaya G (2020). Climate change impacts on water and agriculture sectors in Southern Africa: threats and opportunities for sustainable development. Water.

[3] Hofstra N, Vermeulen LC (2016). Impacts of population growth, urbanisation and sanitation changes on global human Cryptosporidium emissions to surface water. Int J Hyg Environ Health.

[4] Röös E, Bajželj B, Smith P, Patel M, Little D, Garnett T (2017). Greedy or needy? Land use and climate impacts of food in 2050 under different livestock futures. Glob Environ Change.

[5] Food and Agriculture Organization of the United Nations (FAO), International Fund for Agricultural Development (IFAD), United Nations International Children’s Emergency Fund (UNICEF), World Food Programme (WFP) and World Health Organization (WHO) (2018). 2018 The state of food security and nutrition in the world 2018: building climate resilience for food security and nutrition [Internet].

[6] Molotoks A, Smith P, Dawson TP (2021). Impacts of land use, population, and climate change on global food security. Food Energy Secur.

[7] Department of Trade Negotiations (2020). Thailand’s chicken export situation and the utilization of FTAs [Internet].

[8] United States Department of Agriculture (USDA) (2023). Poultry and products annual [Internet].

[9] Office of Agricultural Economics (2023). Thailand foreign agriculture trade statistics [Internet].

[10] Soisontes S (2017). Concerns about sustainability in the poultry industry: a comparative Delphi study in Germany and Thailand. Worlds Poult Sci J.

[11] Sampantamit T, Ho L, Lachat C, Sutummawong N, Sorgeloos P, Goethals P (2020). Aquaculture production and its environmental sustainability in Thailand: challenges and potential solutions. Sustainability.

[12] Kasem S, Thapa GB (2012). Sustainable development policies and achievements in the context of the agriculture sector in Thailand. Sustain Dev.

[13] Boonthongniam N (c2024). Information book on the number of livestock in Thailand for the year 2024 [Internet].

[14] Kornboontritos S (2024). Industry Outlook 2024–2026 chilled, frozen and processed chicken industry [Internet].

[15] Klaharn K, Ngampak R, Chudam Y, Salvador R, Jainonthee C, Punyapornwithaya V (2024). Analyzing and forecasting poultry meat production and export volumes in Thailand: a time series approach. Cogent Food Agric.

[16] Hinjoy S, Thumrin P, Sridet J (2024). An overlooked poultry trade network of the smallholder farms in the border provinces of Thailand, 2021: implications for avian influenza surveillance. Front Vet Sci.

[17] Boonthongniam N (c2022). Information book on the number of livestock in Thailand for the year 2022 [Internet].

[18] Soisontes S (2015). Sustainability in poultry production: a comparative study between Germany and Thailand [dissertation].

[19] Boonthongniam N (c2024). Farmer and livestock information fiscal year 2024 [Internet].

[20] Morris SS, Beesabathuni K, Headey D (2018). An egg for everyone: pathways to universal access to one of nature’s most nutritious foods. Matern Child Nutr.

[21] Thongpalad K, Koirala S, Anal AK (2022). Risk perceptions, on-farm handling, and food safety practices among egg producing farmers in Thailand. J Agribus Dev Emerg Econ.

[22] Observatory of Economic Complexity (OEC) (2024). Bilateral trade profile: eggs – Thailand [Internet].

[23] Statista (2025). Revenue of the egg market in Thailand [Internet].

[24] Pantaweesak N (c2024). Industry outlook 2024: duck meat market analysis [Internet].

[25] Statista (2025). Production volume of duck meat in Thailand from 2014 to 2023 [Internet].

[26] Wongrattanatham P, Pasukphun N (2023). Greenhouse gases emission and environmental costs of fast-food restaurants: a case study in Bangkok, Thailand. EnvironmentAsia.

[27] Marshall BM, Levy SB (2011). Food animals and antimicrobials: impacts on human health. Clin Microbiol Rev.

[28] Diaz-Sanchez S, D’Souza D, Biswas D, Hanning I (2015). Botanical alternatives to antibiotics for use in organic poultry production. Poult Sci.

[29] Singer AC, Xu Q, Keller VDJ (2019). Translating antibiotic prescribing into antibiotic resistance in the environment: a hazard characterisation case study. PLOS ONE.

[30] Iannetti L, Romagnoli S, Cotturone G, Podaliri Vulpiani M (2021). Animal welfare assessment in antibiotic-free and conventional broiler chicken. Animals.

[31] Department of Trade Negotiations, Ministry of Commerce, Thailand (2023). Chicken and chicken products [Internet].

[32] Bracke MBM, Vermeer HM, van Emous RA (2019). Animal welfare regulations and practices in 7 (potential) trade-agreement partners of the EU with a focus on laying hens, broilers and pigs: Mexico, Chile, Indonesia, Australia, New Zealand, Turkey and the Philippines.

[33] Fancher CA, Zhang L, Kiess AS, Adhikari PA, Dinh TTN, Sukumaran AT (2020). Avian pathogenic Escherichia coli and Clostridium perfringens: challenges in no antibiotics ever broiler production and potential solutions. Microorganisms.

[34] Ministry of Natural Resources and Environment (MNRE) (2021). Mid-century, long-term low greenhouse gas emission development strategy: Thailand Submitted under the Paris Agreement United Nations Climate Change.

[35] Ministry of Natural Resources and Environment (MNRE) (2022). Thailand’s long-term low greenhouse gas emission development strategy (revised version).

[36] Office of Natural Resources and Environmental Policy and Planning (ONEP) (2022). Thailand’s second updated nationally determined contribution.

[37] Poore J, Nemecek T (2018). Reducing food’s environmental impacts through producers and consumers. Science.

[38] Ungureanu N, Vlăduţ V, Biriş SŞ (2023). Management of by-products and waste from poultry meat industry. Ann Fac Eng Hunedoara Int J Eng.

[39] Apinda N, Yao Y, Zhang Y (2023). Efficiency of NHEJ-CRISPR/Cas9 and Cre-LoxP engineered recombinant turkey herpesvirus expressing Pasteurella multocida OmpH protein for fowl cholera prevention in ducks. Vaccines.

[40] Linares D, Francisco J, Nogueira L, Caetano M, Pinto E, Mateus MP (2024). Perceptions of insects and algae as alternative protein sources. Proceedings.

[41] Moura MAFE, Martins BA, Oliveira GP, Takahashi JA (2023). Alternative protein sources of plant, algal, fungal and insect origins for dietary diversification in search of nutrition and health. Crit Rev Food Sci Nutr.

[42] Linden J (2023). Thai government supports domestic corn production [Internet]. Feed Strategy.

[43] Linden J (2024). CPF welcomes Thai government’s move on soy imports for feed strategy [Internet]. Feed Strategy.

[44] Jagtap S, Trollman H, Trollman F (2022). The Russia-Ukraine conflict: its implications for the global food supply chains. Foods.

[45] Mordor Intelligence (2024). Thailand feed additives - market share analysis, industry trends & statistics, growth forecasts (2024 – 2029) [Internet]. GII Research.

[46] Pauleta SR, Carepo MSP, Moura I (2019). Source and reduction of nitrous oxide. Coord Chem Rev.

[47] Tilleke & Gibbins (2024). Developments in Thailand’s draft climate change law [Internet].

[48] Panuwet P, Siriwong W, Prapamontol T (2012). Agricultural pesticide management in Thailand: status and population health risk. Environ Sci Policy.

[49] Oryza SM, Wongtangtintharn S, Tengjaroenkul B (2021). Investigation of citric acid by-products from rice produced by microbial fermentation on growth performance and villi histology of Thai broiler chicken (KKU 1). Vet Sci.

[50] Vasquez R, Oh JK, Song JH, Kang D (2022). Gut microbiome-produced metabolites in pigs: a review on their biological functions and the influence of probiotics. J Anim Sci Technol.

[51] Barzegar S, Wu SB, Choct M, Swick RA (2020). Factors affecting energy metabolism and evaluating net energy of poultry feed. Poult Sci.

[52] Pokphand Charoen (2023). CP foods highlights sustainable broiler farming with focus on animal welfare principles at “International Poultry Council 2023” [Internet].

